# Large Fermi Surface of Heavy Electrons at the Border of Mott Insulating State in NiS_2_

**DOI:** 10.1038/srep25335

**Published:** 2016-05-12

**Authors:** S. Friedemann, H. Chang, M. B. Gamża, P. Reiss, X. Chen, P. Alireza, W. A. Coniglio, D. Graf, S. Tozer, F. M. Grosche

**Affiliations:** 1 HH Wills Laboratory, University of Bristol, Bristol, BS8 1TL, UK; 2 Cavendish Laboratory, University of Cambridge, Cambridge, CB3 0HE, UK; 3 Department of Physics, Royal Holloway, University of London, Egham TW20 0EX, UK; 4 Jeremiah Horrocks Institute for Mathematics, Physics and Astronomy, University of Central Lancashire, Preston PR1 2HE, UK; 5 National High Magnetic Field Laboratory, Tallahassee, FL 83810, USA

## Abstract

One early triumph of quantum physics is the explanation why some materials are metallic whereas others are insulating. While a treatment based on single electron states is correct for most materials this approach can fail spectacularly, when the electrostatic repulsion between electrons causes strong correlations. Not only can these favor new and subtle forms of matter, such as magnetism or superconductivity, they can even cause the electrons in a half-filled energy band to lock into position, producing a correlated, or Mott insulator. The transition into the Mott insulating state raises important fundamental questions. Foremost among these is the fate of the electronic Fermi surface and the associated charge carrier mass, as the Mott transition is approached. We report the first direct observation of the Fermi surface on the metallic side of a Mott insulating transition by high pressure quantum oscillatory measurements in NiS_2_. Our results point at a large Fermi surface consistent with Luttinger’s theorem and a strongly enhanced quasiparticle effective mass. These two findings are in line with central tenets of the Brinkman-Rice picture of the correlated metal near the Mott insulating state and rule out alternative scenarios in which the carrier concentration vanishes continuously at the metal-insulator transition.

Prototypical Mott insulators such as V_2_O_3_, NiS, NiO, and NiS_2_ feature singly occupied *d* states with reduced orbital overlap and strong on-site Coulomb repulsion *U*^1^,^2^. The electronic states which would make up a band straddling the chemical potential *μ* if *U* were zero, split at large *U* into a lower and upper Hubbard band, falling on either side of the chemical potential^3^. This gaps out charged excitations and produces an insulating ground state (as sketched in the inset of Fig. 1). The electronic spectrum can be tuned, for instance, by varying the lattice density and thereby the underlying electronic bandwidth *W* with applied pressure, making it possible to close the charge gap and metallize the Mott insulator. The resulting correlated metal displays a narrow peak in the density of states at the chemical potential^4^,^5^ which, within the Brinkman-Rice picture^6^, is associated with long-lived Fermi-liquid quasiparticles.

The transition from the metallic to the insulating state can be realized via a continuous suppression of the quasiparticle weight *Z* and, consequently, a divergence of the effective mass m^*^. In many cases, however, this divergence may be cut off by a first order transition, as is indeed expected in more sophisticated treatments^7^, in particular, if the coupling to the lattice is included^8^. Either way, in the Brinkman-Rice picture the correlated metallic state near the metal-insulator transition (MIT) features an enhanced quasiparticle mass and a large Fermi surface, the volume of which is fixed by Luttinger’s theorem^9^ as identical to that of the corresponding uncorrelated metal. This contrasts fundamentally with alternative scenarios, in which the charge carrier concentration is reduced on approaching the MIT, as might be expected, for instance, in the presence of density-wave order accompanied by Fermi surface reconstruction.

Despite the long history of the Brinkman-Rice picture of a MIT, there is surprisingly little direct experimental evidence for its key predictions – the large Fermi surface expected for half-filled bands and the enhanced effective mass on the threshold of the Mott state. This can be attributed in part to practical difficulties: accessing the threshold of the Mott state requires finely controlled metallization of a Mott insulator either by pressure or chemical substitution. Pressure precludes the use of angular resolved photoemission spectroscopy (ARPES), whereas the disorder associated with substitution seriously hinders high-resolution Fermi surface measurements by quantum oscillatory techniques. Quantum oscillation measurements have been adapted to high pressures in the past but are particularly demanding for MIT, because the supremely important quality of the sample can only be assessed in the metallic phase at high pressure, slowing down the screening process. ARPES, on the other hand, can suffer from complications arising from conducting surface states^10^ and requires exquisite energy resolution to disentangle coherent and incoherent contributions to the spectrum, to determine conclusively the shape of the Fermi surface in 3D, and to determine accurately the effective mass m^*^.

Detailed quantum oscillation studies near a MIT have so far mostly been conducted in two material systems – the superconducting cuprates and the 2D organic charge transfer salts. In the cuprates, however, charge-density-wave reconstruction, superconductivity with high critical fields, and the pseudogap obscure direct access to the large Fermi surface expected to emerge from the doped Mott state^11^. In 2D organic conductors quantum oscillation studies have been limited to samples which are already metallic at ambient pressure^12^, preventing access to the close vicinity of the MIT.

## Results

We study the 3D inorganic Mott insulator NiS_2_, which is free from superconductivity and charge-density-wave reconstruction. NiS_2_ can be tuned into the metallic state by hydrostatic pressure, enabling immediate access to the Fermi surface geometry and quasiparticle mass in close proximity to the MIT. We employ quantum oscillation measurements to focus on the coherent quasiparticle states which lie at the heart of the Brinkman-Rice framework. This approach contrasts with spectroscopic measurements on the Ni(S/Se)_2_ composition series, which give important and comprehensive insight on the summed coherent and incoherent contributions to the spectral function as the MIT is crossed by chemical substitution^13^,^14^,^15^,^16^. It complements recent ARPES results which are interpreted in terms of a progressive reduction of the Fermi velocity on approaching the MIT^17^, but by focusing on the coherent part of the spectrum, quantum oscillation measurements offer definitive and high resolution measurements of the Fermi surface and effective mass in a 3D material.

The pyrite NiS_2_ has long been identified as a prototypical Mott insulator alongside NiS and NiO^2^,^18^,^19^,^20^,^21^,^22^,^23^,^24^,^25^. The sulphur atoms in NiS_2_ form dimers, yielding a valence of 2 for the Ni atoms like in NiO and NiS^25^,^26^. The Ni *d* states are split by the Coulomb interaction into a lower and upper Hubbard band with band edges at −1 eV and 3.5 eV below and above the chemical potential, respectively^25^. Sulphur dimers contribute an antibonding p^*^_σ_ band 1 eV above the chemical potential. Under pressure, the sulphur dimers are rigid but the inter-dimer hopping increases, causing the p^*^_σ_ band to broaden and eventually to connect with the lower Hubbard band, inducing metallization. Whereas megabar pressures are required to metallize NiO, rendering quantum oscillation measurements impossible with current techniques, the tunability of the sulfur dimer-derived bands in NiS_2_ reduces the metallization pressure to about 3 GPa, where the key impediment to high pressure quantum oscillation measurements – pressure inhomogeneity caused by the pressure medium – is well understood and under control.

**Figure 1:**
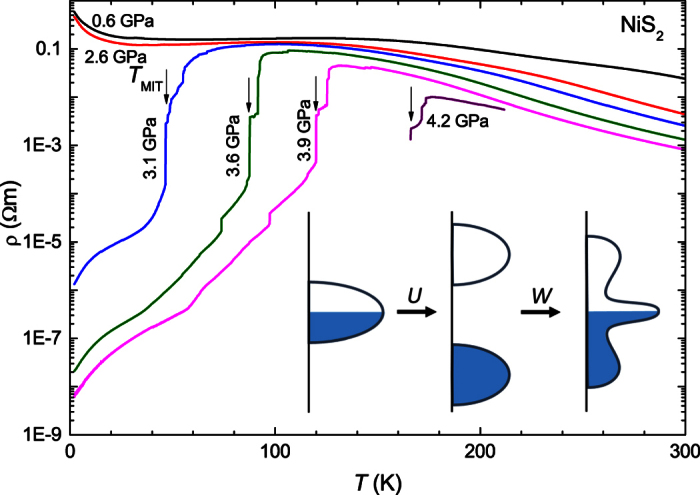
Resistivity of NiS_2_ under pressure. For pressures larger than 2.6 GPa, a rapid drop in the resistivity is observed at a temperature *T*_MIT_, defined from the steepest slope. (Inset) Schematic representation of the formation of upper and lower Hubbard bands for large Coulomb interaction *U*, and of the emergence of a coherent quasiparticle peak at the chemical potential, when the bandwidth *W* is increased, for instance by tuning the lattice density under pressure^3^,^4^,^5^.

Sulphur vacancies are known to compromise crystal quality in NiS_2_. Employing a Te-flux growth technique^27^ we obtained crystals with ultra-low sulphur vacancy concentration (cf. Supplementary information I). Our high-quality single crystals of NiS_2_ display clear insulating behavior at low pressure (Fig. 1). We observe the magnetic transition into the weak ferromagnetic state at *T*_WF_ = 30 K at ambient pressure in agreement with previous results^28^ (cf. Supplementary information II).

The application of hydrostatic pressure in our liquid-medium patterned-anvil cell (cf. Materials and Methods) first yields very little change at low temperature *T* (Fig. 1). A drastic change is observed at a pressure of 3 GPa: Here, the low-temperature resistivity is reduced by many orders of magnitude, signaling the MIT. The steps in the resistivity curves when passing through the MIT may indicate that the volume contraction on metallization^29^ of consecutive parts of the sample affects the stress acting on the remaining sample, in particular considering that the pressure medium is frozen at these temperatures. This can cause different parts of the sample to undergo the MIT at different temperatures, but pressure homogeneity is restored at low temperature, when the sample is fully metallic. The metallic state below the MIT temperature *T*_MIT_ is recognized from the positive slope of the resistivity dρ/d*T* > 0. Increasing the pressure beyond 3 GPa results in a further reduction of the low-temperature resistivity and a shift of *T*_MIT_ to higher temperatures.

**Figure 2:**
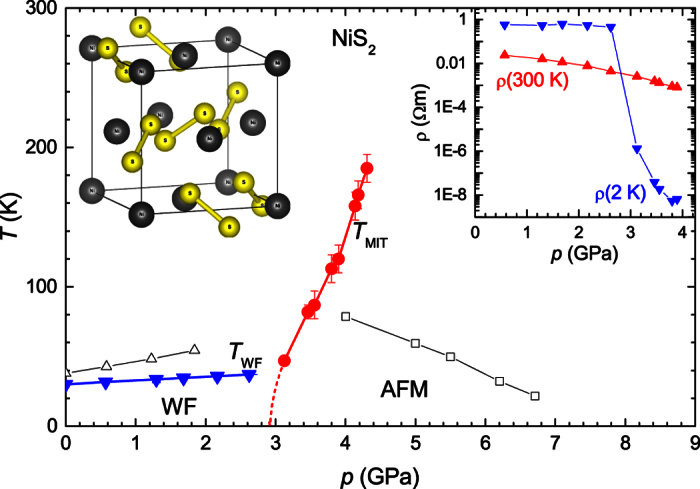
High-pressure phase diagram of NiS_2_. Solid circles and triangles represent the transition temperatures of the MIT and ferromagnetic order (cf. Supplementary information II) as extracted from resistivity, respectively. Open squares and triangles reflect the magnetic transition temperatures in the metallic and insulating state from Refs 30,28, respectively. Left inset shows the pyrite structure of NiS_2_ with the sulphur dimers indicated. Right inset shows the evolution of room-temperature (red triangles) and low-temperature (blue triangles) resistivity.

Based on the resistivity measurements we can construct the *T*-*p* phase diagram for our NiS_2_ samples (Fig. 2). We find *T*_WF_ and *T*_MIT_ very similar to previous high pressure transport studies^28^,^30^. The slightly higher pressure scale is attributed to the absence of sulphur vacancies in our samples (cf. Supplementary information I). We followed the MIT up to 4.3 GPa and 185 K. The transition becomes less distinct towards the critical end point of the first order MIT line at around 200 K^30^. The antiferromagnetic transition is not reliably resolved in the temperature dependence of the resistivity, in accordance with previous reports studying both the insulating state as well as the metallic state^30^,^31^.

The abrupt decrease of the residual resistivity by 6 orders of magnitude on crossing the MIT by applied pressure is highlighted in the inset of Fig. 2. This is followed by a further decrease of more than 2 orders of magnitude in the metallic phase. Importantly, with *ρ*_0_ falling below 1 μΩ cm, high-resolution Fermi surface studies with quantum oscillation measurements become possible.

**Figure 3:**
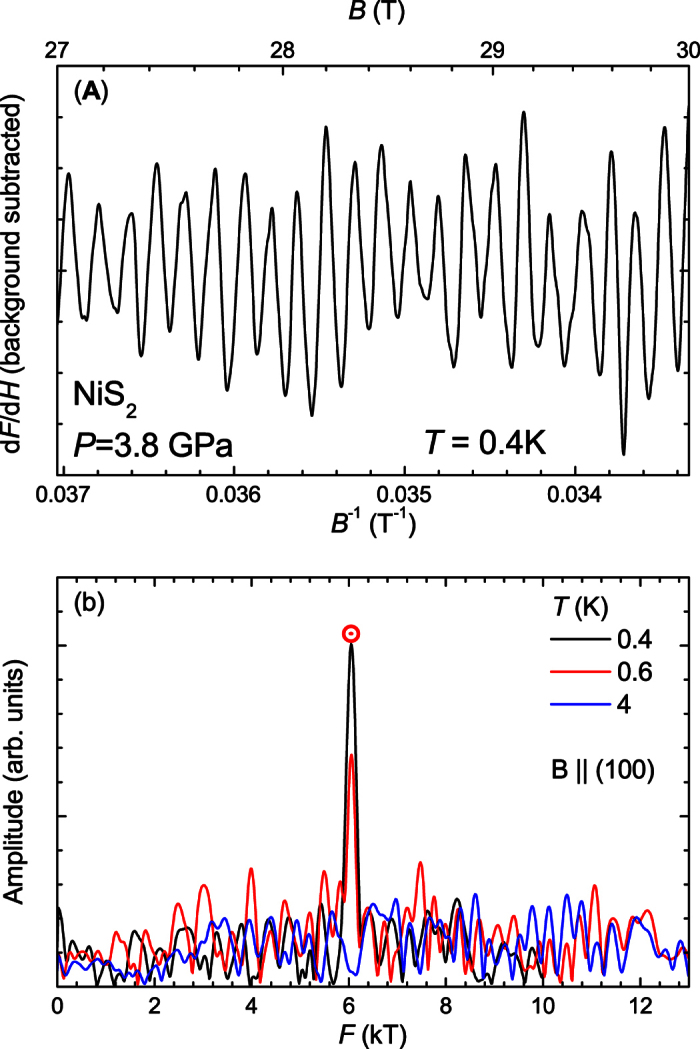
Quantum oscillations in the metallic phase of NiS_2_. (**A**) We analyze the derivative d*F*/d*H* of the TDO frequency *F* with respect to field *H*. Numerical differentiation used locally fitted polynomials. A smooth background has been subtracted. Plotting against inverse magnetic field reveals the characteristic periodicity in 1/*H* of quantum oscillations. Data obtained from several sweeps with different sweep rates are averaged thus ruling out parasitic signals as a source of 1/*H* periodicity. (**B**) Power spectra of the Fourier transformed signal were obtained at several temperatures. The magnetic field was oriented along the crystallographic (100) direction within the cubic unit cell notation.

We measure quantum oscillations in the resistivity by a contactless tunnel-diode-oscillator (TDO) method for increased resolution and to circumvent difficulties associated with making contacts to the sample. A single crystal NiS_2_ sample is placed inside the micro-coil mounted in the hole of a gasket between two opposing moissanite anvils^32^ (see Materials and Methods). Fig. 3(A) displays the quantum oscillation signal observed at a pressure of 3.8 GPa, where the residual resistivity is well below 1 μΩ cm. We find clear oscillations at high fields beyond 25 T. The Fourier transform in Fig. 3(B) identifies the oscillation frequency to be 6 kT.

**Figure 4:**
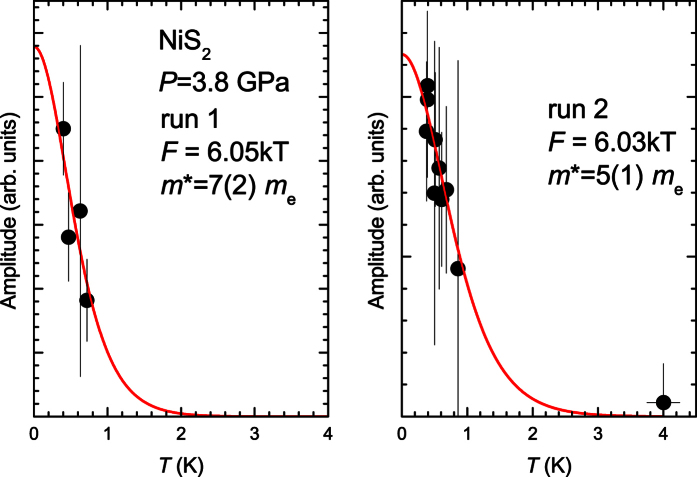
Determination of the effective mass. The temperature dependence of the quantum oscillation amplitude (solid circles) is fitted with the Lifshitz-Kosevich form for two subsequent runs (solid red line). Vertical lines reflect standard errors estimated from background in the Fourier spectrum close to 6 kT (cf. Fig. 3).

The temperature dependence of the quantum oscillation amplitude can be used to extract the quasiparticle mass. This is illustrated for two subsequent runs in Fig. 4. Best fits to the data yield effective masses of m^*^ = 5(1) *m*_e_ and m^*^ = 7(2) *m*_e_, respectively. Combining the two datasets we conclude the quasiparticle mass to be m^*^ = 6(2) *m*_e_. Despite the uncertainty in m^*^, our measurement provides high significance for the comparison with band structure calculations, to which we now turn.

**Figure 5:**
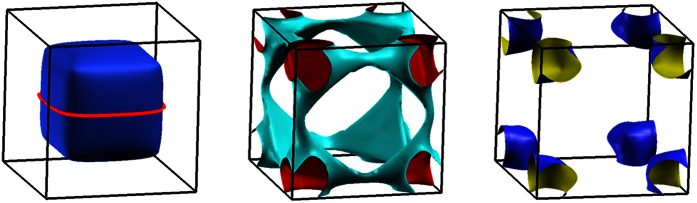
Calculated Fermi surface. The Fermi surface was determined from band structure calculations using the lattice parameters and atomic positions as determined by our x-ray diffraction (see Supplementary information I). Besides the major sheets depicted here we find two small pockets in the Brillouin-zone corners. The solid red line on the first sheet represents its belly orbit.

The electronic structure of metallic NiS_2_ has been calculated within the generalized gradient approximation, neglecting strong correlations. We use the simple cubic crystal lattice with lattice constants estimated for our crystals at 3.8 GPa (Supplementary information III). At the pressures studied, NiS_2_ is suggested to be antiferromagnetic at low temperature^30^. High pressure diffraction studies have so far not resolved the spin structure in metallic NiS_2_^29^,^33^, but neutron diffraction studies in the Ni(S/Se)_2_ composition series (e.g. Ref. 34) suggest that the antiferromagnetic ordering wave vectors in the metallic state are (1 0 0) and its symmetry-related equivalents, as in ambient pressure, insulating NiS_2_ at intermediate temperatures. In the latter case, a type 1 antiferromagnetic structure has been proposed^35^, for which the magnetic unit cell is the same as the structural unit cell and no Fermi surface reconstruction would necessarily be expected. Our calculation predicts a metallic ground state, in good agreement with previously published local density approximation results^25^,^36^,^37^,^38^. Modelling the insulating state would require including onsite Coulomb repulsion in the spirit of the Mott-Hubbard model^39^. Here, we compare the calculated metallic Fermi surface with our measurements in the metallic state at high pressure. The major Fermi surface sheets obtained in the band structure calculations are presented in Fig. 5: they consist of a large “Cube”-like pocket in the center of the Brillouin zone, a network connected along the edges of the Brillouin zone, and several closed pockets in the corner (only the biggest of which is presented in Fig. 5). From the predicted Fermi surface we can identify the most likely orbit to produce quantum oscillations: The small curvature on the “Cube” makes it a strong candidate. Indeed, the predicted frequency of 6.3 kT for the belly orbit (highlighted in Fig. 5) shows excellent agreement with the observed quantum oscillation frequency of 6 kT (cf. Table 1). In particular, it is the only orbit large enough to yield a frequency above 3 kT (cf. Supplementary information III).

## Discussion

Our quantum oscillation results demonstrate unequivocally that the correlated metallic state on the threshold of Mott localization in NiS_2_ is characterized by a large Fermi surface consistent with a high carrier concentration. This contrasts fundamentally with the proposal based on electrical transport measurements^40^ that the carrier concentration and thereby the Fermi surface volume decreases continuously on approaching the MIT. This discrepancy may be attributed to phase separation into metallic and insulating domains near the MIT, which would make transport data an unreliable probe of the true metallic state as probed by quantum oscillation measurements.

Whereas the predicted and observed frequencies are in excellent agreement we find a strong deviation between the predicted and observed effective mass (Table 1). The strong mass enhancement by nearly one order of magnitude observed in high pressure NiS_2_ can be attributed to the strong correlations expected within the Brinkman-Rice picture^6^. This mass enhancement should be accompanied by a similarly large enhancement of the Sommerfeld coefficient of the specific heat capacity γ over the band structure value. A roughly four-fold increase of γ has indeed been observed in the composition series Ni(S_1−x_Se_x_)_2_ on approaching the Mott transition from the metallic side^40^,^41^. The mass enhancement is consistent, also, with the narrow band features observed in ARPES studies of metallic members of the Ni(S_1−x_Se_x_)_2_ series^13^. Together with the cross-sectional area determined from the 6 kT orbit and assuming a spherical Fermi surface geometry, the measured mass corresponds to a Fermi velocity *v*_F_~0.4 eV Å/ħ, comparable to results from ARPES measurements at the corresponding doping of x = 0.5 (Ref. 17). It will be very interesting to follow the pressure dependence of the quasiparticle mass in further quantum oscillation measurements, which may reveal a further increase of m* on approaching the MIT. The discontinuous volume contraction of the order of 1% observed on crossing the MIT at high pressure with temperature x-ray diffraction^29^, suggests that the MIT itself is first order at low temperature, which may interrupt a mass divergence at a level not far above our measured m*.

High quality crystals of NiS_2_ have enabled a fresh look at the Mott transition in this prototypical material using a novel combination of high-pressure techniques. Our quantum oscillation measurements unambiguously show that coherent quasiparticles with a large Fermi surface and significant mass enhancement exist near the border of Mott localization in pressure metallized NiS_2_. These findings offer a direct view on the emergence of a heavy Fermi liquid on the metallic side of the Mott transition and motivate further, detailed high pressure quantum oscillation studies in NiS_2_ and other Mott insulators.

## Materials and Methods

**Single crystals** of NiS_2_ were grown from Te-flux as described earlier^27^. Sulphur occupation was determined from single-crystal x-ray diffraction as detailed in Supplementary information I and are found to be in very good agreement with estimates based on the temperature dependence of the resistivity^22^.

**Resistivity studies** were conducted in an alumina Bridgman anvil cell with patterned anvils^42^ with Flourinert as pressure transmitting medium. The superconducting transition temperature of a lead sample was used to determine the pressure at low temperatures.

**Quantum oscillation measurements** used the contactless tunnel-diode-oscillator (TDO) technique, which involves a parallel inductor-capacitor oscillator sustained by a tunnel diode. The inductor in the experimental setup was a five turn cylindrical coil with an inner diameter of 200 μm wound with a 15 μm insulated wire. For high pressure measurement, the coil is positioned in a 300 μm diameter hole in a BeCu gasket mounted between the anvils of a Moissanite anvil pressure cell. An oriented single crystal sample of NiS_2_ was placed in the coil. Changes in the sample resistivity in its metallic state are detected as proportional changes in the TDO resonance frequency.

A 4:1 mixture of methanol-ethanol was used as pressure transmitting medium for highly hydrostatic conditions. Sample pressure at low temperature was determined via ruby fluorescence. Quantum oscillation measurements were carried out at the NHMFL Tallahassee, with the TDO oscillating at ~250 MHz, in a top-loading ^3^He cryostat in magnetic fields up to 31 T.

**Data availability**: All data needed to evaluate the conclusions in the paper are present in the paper, the Supplementary Materials and the Data repository at the University of Cambridge and can be download from https://www.repository.cam.ac.uk/handle/1810/255127. Additional data related to this paper may be requested from the authors.

## Additional Information

**How to cite this article**: Friedemann, S. *et al.* Large Fermi Surface of Heavy Electrons at the Border of Mott Insulating State in NiS_2_. *Sci. Rep.* **6**, 25335; doi: 10.1038/srep25335 (2016).

## Supplementary Material

Supplementary Information

## Figures and Tables

**Table 1:** Comparison of band structure calculation and experiment.

NiS_2_	Calc.	Exp.
*F* (kT)	6.3	6.03
m^*^ (*m*_e_)	0.8	6(2)

Frequency and effective mass calculated for the belly orbit of the “Cube” Fermi surface sheet are compared to those obtained from our QO measurements.
