# Erratum: Large Fermi Surface of Heavy Electrons at the Border of Mott Insulating State in NiS_2_

**DOI:** 10.1038/srep36690

**Published:** 2016-11-10

**Authors:** S. Friedemann, H. Chang, M. B. Gamża, P. Reiss, X. Chen, P. Alireza, W. A. Coniglio, D. Graf, S. Tozer, F. M. Grosche

Scientific Reports 6: Article number: 2533510.1038/srep25335; published online: 05 12 2016; updated: 11 10 2016

The Supplementary Information file originally published with this Article contained errors.

In Table S1, the 2θ_min_/2θ_max_ values “6.2/53.5” were incorrectly given as “0.076/0.013”.

In the ‘calculation’ column of Table S2, the S value “0.1053” was incorrectly given as “0.10595” and the a(Å) value “5.610” was incorrectly given as “5.698”.

In addition, in the legend of Table S2.

“**Lattice parameters, atomic positional and displacement parameters for NiS_2_** (Note: B_12_ = B_13_ = B_23_ and B_11_ = B_22_ = B_33_ for 4*a* site, B_13_ = B_23_ = −B_12_ and B_11_ = B_22_ = B_33_ for 8*c* site). The structural data on our Te-flux grown crystals are compared to those for crystals grown with vapor transport technique (VT)^2,3^”.

now reads:

“**Lattice parameters, atomic positional and displacement parameters for NiS_2_** (Note: B_12_ = B_13_ = B_23_ and B_11_ = B_22_ = B_33_ for 4*a* site, B_13_ = B_23_ = −B_12_ and B_11_ = B_22_ = B_33_ for 8*c* site). The structural data on our Te-flux grown crystals are compared to those for crystals grown with vapor transport technique (VT)^2,3^. The last column shows the values used for the band structure calculation taking into account the compression at high pressures as described in the main text”.

These errors have been corrected in the Supplementary Information that now accompanies the Article.

