# Supplementary material for: Large Fermi Surface of Heavy Electrons at the Border of Mott Insulating State in NiS2
Source: Sci Rep. 2016 May 12;6:25335. doi: 10.1038/srep25335 (PMC4865941; doi:10.1038/srep25335)
Supplement: Supplementary Information [file srep25335-s1.pdf]

Supplementary Materials to

"Large Fermi Surface of Heavy Electrons at the Border of Mott Insulating State in NiS_2"

S. Friedemann,[1*] H. Chang,[2] M. B. Gamża,[3,4] P. Reiss,[2] X. Chen,[2] P. Alireza,[2] W. A. Coniglio[5], D. Graf,[5] S. Tozer,[5] and F. M. Grosche[2 *]

[1] HH Wills Laboratory, University of Bristol, Bristol, BS8 1TL, UK.

[2] Cavendish Laboratory, University of Cambridge, Cambridge, CB3 0HE, UK.

[3] Department of Physics, Royal Holloway, University of London, Egham TW20 0EX, UK.

[4] Jeremiah Horrocks Institute for Mathematics, Physics and Astrophysics, University of Central Lancashire, Preston PR1 2HE, UK.

[5] National High Magnetic Field Laboratory, Tallahassee, FL 83810, U.S.A.

[*]Corresponding authors: SF – Sven.Friedemann@bristol.ac.uk, FMG – fmg12@cam.ac.uk

Supplementary Information I: Characterization of high-quality NiS_2 single crystals

Structural characterization of our crystals was carried out at room temperature using an Xcalibur E Single Crystal Diffractometer. Details concerning data collection and handling are summarized in table S1. Powder x-ray diffraction measurements were performed using a Bruker D2 Phaser diffractometer (Cu K-α radiation, 2θ interval of 3–80 degrees, step size of 0.018 degree, expose time of 4 hours) on powder prepared by grinding crystals with Si as an internal standard. Structure refinements based on both single crystal diffraction data and powder diffraction patterns were carried out using the Jana2006 program [1]. X-ray diffraction measurements confirm that our crystals adopt the Pa-3 space group, the pyrite–type structure [2,3]. The relevant crystallographic information is listed in table S1. Consistent results were obtained for several crystals across the batch. According to the single crystal X-ray diffraction study, our crystals are stoichiometric and have both Ni and S sites fully occupied (Table S2). Refinements with initial values of occupancies set as 0.95 converged with negligible populations of vacancies of less than ~0.1% for both crystallographic sites. This needs to be contrasted with previous studies on crystals grown using vapor transport technique, for which ~2% of S sites were found to be vacant [2,3]. Furthermore, our diffraction measurements performed on a number of crystals with typical dimensions of 0.1–0.2 mm showed mosaicities of 0.4–0.5 degrees. Such low values of the mosaicity parameter indicates a high degree of perfection of the crystal lattice.

The temperature-pressure phase diagram as constructed from our resistivity measurements is very similar to results published earlier [4,5]. However, we find a slightly larger critical pressure of ~2.9 GPa compared to 2.5 GPa reported in earlier studies. The full phase diagram can be scaled between our studies and the earlier results on vapor-transport grown samples. This is illustrated in Fig. S1: Both the MIT and the magnetic transition coincide for our samples and the vapor-transport samples, when a scaling factor of 1.28 is applied to the pressure axis. This indicates a smaller compressibility of our crystals, which we attribute to the absence of vacancies.

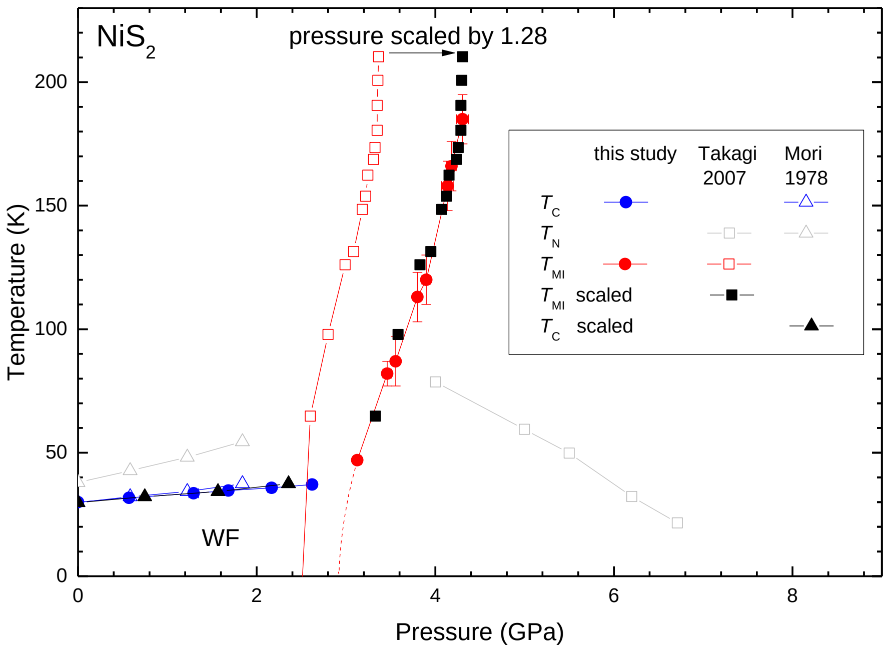

Figure S1: Phase diagram of NiS_2 – Comparison with vapor-transport grown samples. The MIT transition temperature T_{MIT} and the transition temperature T_{WF} into the weak ferromagnetic state (WF) of vapor-transport grown samples [4,5] can be scaled to our results using a factor of 1.28 for the pressure axis as illustrated with black solid squares (T_{MIT}) and triangles (T_{WF}).

Structure type	pyrite
Space group	Pa-3 (No. 205)
Formula units/cell	4
Diffraction system	Xcalibur E, four-circle Kappa
	Sapphire CCD Detector (Xcalibur)
Radiation, λ (Å)	Mo Kα, 0.71073
Temperature (K)	295(5)
Range in h,k,l	±12, ±12, ±12
R(eqv)/R(σ)	0.076/0.013
$2\theta_{min}/2\theta_{max}$	0.076/0.013
Observation criteria	F(hkl) > 3.00 σ(F)
Resolution d(Å)	0.45
Absorption coefficient	Face-based, analytical [6]
Absorption coefficient	20.670
N(hkl) measured	23925
N(hkl) unique	364
Extinction method	isotropic type 2 correction [7]
Extinction coefficient	8200(300)
Goodness-of-fit (GOF)	1.13
R	1.59%
wR	1.83%

Table S1. Crystallographic data for NiS_2.

NiS_2		Te-flux					VT	calculation
Atom	Wyckoff site	x	B_{11}	B_{13}	B_{iso}	Occupancy	Occupancy	x
Ni	4*a* (0, 0, 0)	-	0.517(4)	0.011(2)	0.517(2)	1	1	-
S	8*c* (*x*, *x*, *x*)	0.10531(2)	0.464(4)	0.017(2)	0.464(2)	1	0.98	0.10595
a(Å)		5.6893(5)[a]					5.689	5.698

[a]powder data

Table S2. Lattice parameters, atomic positional and displacement parameters for NiS_2 (Note: B_{12}=B_{13}=B_{23} and B_{11}=B_{22}=B_{33} for 4*a* site, B_{13}=B_{23}=-B_{12} and B_{11}=B_{22}=B_{33} for 8*c* site). The structural data on our Te-flux grown crystals are compared to those for crystals grown with vapor transport technique (VT) [2,3].

Supplementary Information II: Magnetic ground state in the insulating phase

A sizeable magnetization develops in our crystals below a transition temperature T_{WF}, suggesting a ferromagnetic component, in agreement with previous data. This transition can be interpreted as spin canting within an overall antiferromagnetic state and manifests in the resistivity as a kink, which we can follow under pressure in the insulating phase as demonstrated in Fig. S2(B). The pressure dependence as shown in Fig. S1 agrees with published data as described in Supplementary information I.

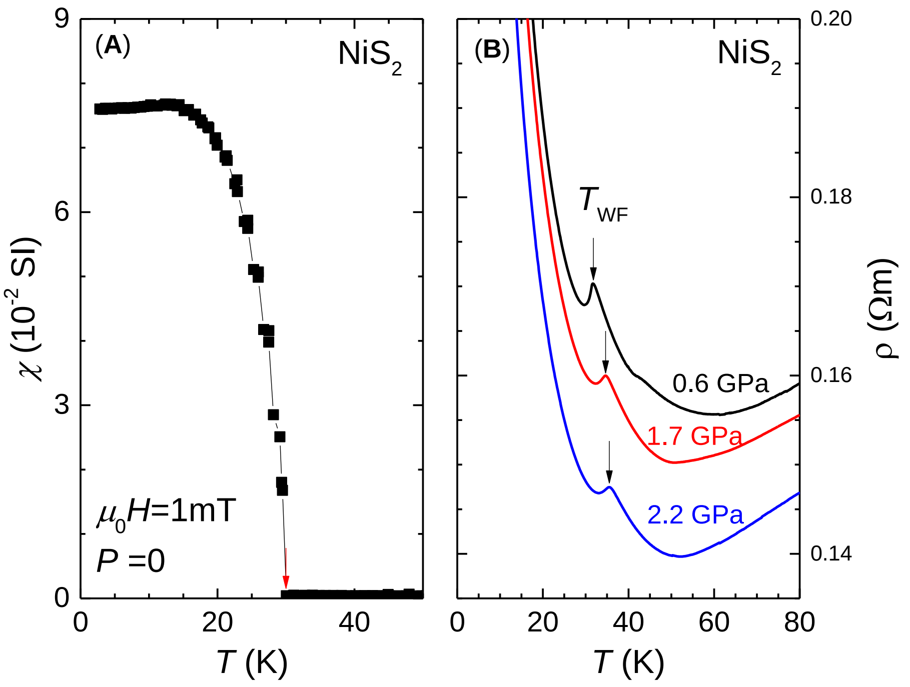

Fig. S2. Ferromagnetic transition in the insulating phase. (A) Ambient pressure magnetization measurements show the ferromagnetic ground state below T_{WF} indicated by the arrow. (B) Resistivity measurements show the pressure dependency of T_{WF} within the insulating phase.

Supplementary Information III: Band structure calculation

Band structure calculations were performed using the WIEN2k density functional code [8]. Band energies were obtained on a 100000k-point mesh in the first Brillouin zone utilizing the generalized gradient approximation to the exchange correlation potential [9].

Relaxing the crystal parameters yielded good agreement with the experimentally observed values for both the lattice parameter and the sulphur position, as shown in table S2. This supports the validity of using the experimental structural parameters for our band structure calculations. We use the experimental lattice parameters determined for our sample and take into account a pressure-induced reduction of the lattice constant by 1.4% at 3.8 GPa. This reduction includes a correction for the reduced compressibility as discussed in Supplementary Information I over the reduction of 1.5% in Ref. [10]. We find 5 bands crossing the Fermi energy with both nickel and sulphur character. The Fermi surfaces are visualized in Fig. 5 with the XCrySDen program [11]. Extremal orbits and effective masses were extracted using the SKEAF algorithm capable of finding extended orbits across several reciprocal unit cells [12].

All orbits predicted for the orientation of the crystal used in our quantum oscillation experiment are listed in table S3. Only the belly orbit of the “Cube” is in agreement with the observed frequency of 6 kT, all other orbits are below 3 kT.

We investigated the effect of a possible collinear type-I antiferromagnetic spin structure on the Fermi surface geometry within Wien2K. This causes changes to all the major sheets, including a distortion of the central “Cube”, but the frequency and mass of the main cube orbit remains nearly unaffected apart from a slight splitting, which is below our frequency resolution.

Band	Orbit	F (kT)	m^* (m_e)
56	56a	6.3	0.8
57	57a	0.04	0.3
	57b	2.8	3.8
58	58a	2.5	3.6
	58b	3.0	1.8
59	59a	0.6	1.3
60	60a	0.6	1.3
exp		6.03	6(2)

Table S3. Predicted frequencies on all Fermi surface sheets.

References

1. Petricek, V., Dusek, M. & Palatinus, L. Crystallographic Computing System JANA2006: General features. *Zeitschrift für Krist. - Cryst. Mater.* **229,** (2014).

2. Yao, X. & Honig, J. M. Growth of nickel dichalcogenides crystals with pyrite structure from tellurium melts [NiS_2, $NiS_{2-x}Se_x$ (x <= 0.7)]. *Mater. Res. Bull.* **29,** 709–716 (1994).

3. Kwizera, P., Dresselhaus, M. & Adler, D. Electrical properties of $NiS_{2-x}Se_x$. *Phys. Rev. B* **21,** 2328–2335 (1980).

4. Mori, N. & Watanabe, T. Pressure effects on the magnetic transition temperatures of NiS_2. *Solid State Commun.* **27,** 567–569 (1978).

5. Takeshita, N. *et al.* Quantum criticality and disorder in the antiferromagnetic critical point of NiS_2 pyrite. *arXiv:0704.0591v1 [cond-mat.str-el]* (2007).

6. Clark, R. C. & Reid, J. S. The analytical calculation of absorption in multifaceted crystals. *Acta Crystallogr. Sect. A Found. Crystallogr.* **51,** 887–897 (1995).

7. Becker, P. J. & Coppens, P. Extinction within the limit of validity of the Darwin transfer equations. I. General formalism for primary and secondary extinction and their applications to spherical crystals. *Acta Crystallogr. Sect. A* **30,** 129–147 (1974).

8. Blaha, P., Schwarz, K., Madsen, G., Kvasnicka, D. & Luitz, J. WIEN2k. (2014).

9. Perdew, J. P., Burke, K. & Ernzerhof, M. Generalized Gradient Approximation Made Simple. *Phys. Rev. Lett.* **77,** 3865–3868 (1996).

10. Feng, Y., Jaramillo, R., Banerjee, A., Honig, J. M. & Rosenbaum, T. F. Magnetism, structure, and charge correlation at a pressure-induced Mott-Hubbard insulator-metal transition. *Phys. Rev. B* **83,** 35106 (2011).

11. Kokalj, A. XCrySDen—a new program for displaying crystalline structures and electron densities. *J. Mol. Graph. Model.* **17,** 176–179 (1999).

12. Rourke, P. M. C. & Julian, S. R. Numerical extraction of de Haas - van Alphen frequencies from calculated band energies. *Comput. Phys. Commun.* **183,** 16 (2008).
